# An online, public health framework supporting behaviour change to reduce dementia risk: interim results from the ISLAND study linking ageing and neurodegenerative disease

**DOI:** 10.1186/s12889-023-16805-2

**Published:** 2023-09-29

**Authors:** Larissa Bartlett, Aidan Bindoff, Kathleen Doherty, Sarang Kim, Claire Eccleston, Alex Kitsos, Eddy Roccati, Jane Alty, Anna E. King, James C. Vickers

**Affiliations:** 1 Wicking Dementia Research and Education Centre, University of Tasmania, Hobart, Australia; 2 Australian Institute of Health and Welfare, Canberra, Australia

**Keywords:** Dementia risk, Public health, Online, Prospective cohort, Health behaviour change

## Abstract

**Background:**

Unmanaged cardiometabolic health, low physical and cognitive activity, poor diet, obesity, smoking and excessive alcohol consumption are modifiable health risk factors for dementia and public health approaches to dementia prevention have been called for. The Island Study Linking Ageing and Neurodegenerative Disease (ISLAND) is a dementia prevention public health study examining whether improving knowledge about modifiable dementia risk factors supports behaviour changes that reduce future dementia risk.

**Methods:**

Residents of Tasmania, Australia, aged 50 + years who joined the 10-year ISLAND study were asked to complete annual online surveys about their knowledge, motivations and behaviours related to modifiable dementia risk. ISLAND included two knowledge-based interventions: a personalised Dementia Risk Profile (DRP) report based on survey responses, and the option to do a 4-week Preventing Dementia Massive Open Online Course (PDMOOC). Longitudinal regression models assessed changes in the number and type of risk factors, with effects moderated by exposures to the DRP report and engagement with the PDMOOC. Knowledge and motivational factors related to dementia risk were examined as mediators of risk behaviour change.

**Results:**

Data collected between October 2019 and October 2022 (n = 3038, av. 63.7 years, 71.6% female) showed the mean number of modifiable dementia risk factors per participant (range 0 to 9) reduced from 2.17 (SD 1.24) to 1.66 (SD 1.11). This change was associated with the number of exposures to the DRP report (p = .042) and was stronger for PDMOOC participants (p = .001). The interaction between DRP and PDMOOC exposures yielded a significant improvement in risk scores (p = .004). The effect of PDMOOC engagement on behaviour change was partly mediated by increased knowledge (12%, p = .013). Self-efficacy enhanced the effect of knowledge on behaviour change, while perceived susceptibility to dementia mitigated this relationship.

**Conclusions:**

The ISLAND framework and interventions, a personalised DRP report and the four-week PDMOOC, work independently and synergistically to increase dementia risk knowledge and stimulate health behaviour change for dementia risk reduction. ISLAND offers a feasible and scalable public health approach for redressing the rising prevalence of dementia.

**Supplementary Information:**

The online version contains supplementary material available at 10.1186/s12889-023-16805-2.

## Introduction

Dementia is a debilitating condition caused by neurodegenerative disorders such as Alzheimer’s Disease that affect brain structure and function. Evidence shows that population growth and longer life expectancies are driving a global rise in dementia incidence [1], with current estimates projecting a three-fold increase from 57 million cases in 2019 to 152 million cases in 2050 [2]. While the pursuit of effective treatment targets, technologies and drugs is progressed, a focus on prevention and early intervention has been called for both globally [3], and in Australia [4].

Findings from the 2020 Lancet Commission report on Dementia Prevention, Intervention and Care [5] show that as much as 40% of the risk of developing dementia is attributable to 12 health-related behaviours and lifestyle factors. Modifiable dementia risk factors include unmanaged hypertension, cholesterol levels and diabetes, low physical and cognitive activity, poor diet, obesity, smoking and excessive alcohol consumption. These risk factors are health and lifestyle-related behaviours that put people at risk of poor health through cardiometabolic diseases as well as dementia, and there are established, clinically relevant cut points at which individuals can be classified as healthy, at-risk or unhealthy [6]. The World Health Organisation suggests interventions that support shifting from high to low risk on any one risk domain can potentially lower an individual’s future risk of dementia and other chronic diseases [7]. Prospective cohort-based intervention studies that aim to increase knowledge about, and catalyse changes in modifiable dementia risk factors offer a potentially effective public health approach to reducing the rising prevalence of dementia [8, 9].

The leading challenge for public health campaigns aiming to change health risk behaviours lies in effectively translating evidence into knowledge, and knowledge into action-taking [10–12]. This challenge is evident in variable results from dementia risk reduction initiatives. The findings reported by Heger, Kohler [13] show no change in dementia risk knowledge was observed following a health promotion campaign that used mass media to disseminate information about brain health. In contrast, Choi, La Monica [14] found that when this risk information was presented as a personalised report of dementia risk behaviours, participants’ accuracy in identifying factors that might affect their chances of developing dementia increased, and their perceptions of personal risk were revised. Similarly, in a study of Dutch adults [15], general knowledge was insufficient for driving health behaviour change, but awareness of one’s own perceived personal risk was considered instrumentally helpful.

Health behaviour change models typically present a progressive pathway from lack of knowledge to motivation to action [16, 17]. According to the Health Beliefs Model (HBM) [16, 17], knowledge influences health perceptions and drives health behaviours. The term knowledge is used throughout this paper to refer to the ability to understand basic health information and make appropriate health decisions [18]. Recognising, recalling and appraising the accuracy and relevance of information about risk factors for dementia are important aspects of dementia risk knowledge [13, 14]. In addition to knowledge, there are several motivational factors in the HBM that influence the likelihood of health behaviour change [17]. These include threat-based constructs such as the perceived severity of and susceptibility to developing disease and disability; reward-based constructs including perceived benefits and barriers associated with changing risk behaviours; and personal resources such as self-efficacy, cues to action and general health intentions [17, 19].

The Island Study Linking Ageing and Neurodegenerative Disease (ISLAND) is a large, prospective dementia prevention public health study [20]. Established in 2019 in Tasmania, Australia, ISLAND will run to at least 2029 with the objective of improving self-management of modifiable dementia risk factors and reducing the future risk of dementia in a large sample of community-dwelling older adults. The core hypothesis for ISLAND was premised on behaviour change theory and supporting evidence that knowing one’s own health risk profile increases the likelihood of taking action to improve and maintain good health [21, 22], and that lack of health knowledge is a key barrier to making lifestyle adjustments to improve brain health [23, 24]. According to Nutbeam [25], health education is an important public health intervention approach as it increases actionable knowledge (or health literacy), which supports healthy lifestyles and appropriate health services access, and is associated with reduced morbidity, disability and avoidable mortality.

Two core knowledge-based interventions were implemented in the ISLAND protocol. All participants were provided a personalised Dementia Risk Profile (DRP) report [26] which presents, in traffic light format, individuals’ risk level (low, medium or high) across nine domains of modifiable dementia risk. ISLAND participants were also invited to complete the 4-week Preventing Dementia Massive Open Online Course (PDMOOC) [27]. These two ISLAND interventions directly address calls by the World Health Organisation (WHO) [7] and the Australian Institute of Health and Welfare [30] for public health approaches to prevent dementia through risk reduction, and recommendations in the WHO Jakarta Declaration, to prioritise empowering people to take responsibility for their own long-term health [31].

The current study examines the effects of the ISLAND intervention framework on the number of modifiable risk factors for dementia (Aim 1). We tested two a-priori hypotheses under Aim 1. First (H1), that time in ISLAND and the number of exposures to the DRP report between baseline and 2022 would be associated with (a) a reduction in the reported number of dementia risk factors, (b) increased dementia risk knowledge and (c) stronger motivations to change behaviours to reduce dementia risk. The second hypothesis under Aim 1 (H2) was that improvements observed in dementia risk behaviours, knowledge and motivations would be stronger for participants who undertook the PDMOOC than those who did not. Further, we used the categorical DRP data to gain a preliminary understanding of which, if any, of the individual risk domains in the DRP demonstrated the most change.

Next, to test the ISLAND knowledge-to-behaviour-change hypothesis in line with the HBM approach, we examined the degree to which a reduction in the number of dementia risk factors was attributable to changes in dementia risk knowledge and motivational factors (Aim 2). We hypothesized that increasing the ability to identify misconceptions about modifiable dementia risk (knowledge) through exposure to the DRP report and the PDMOOC would support positive behaviour change (H3). We then explored the mediating influence of changes in threat-based motivation (perceived susceptibility to dementia) and changes in resource-based motivation (self-efficacy) on the knowledge-to-behaviour-change path.

## Methods

### Recruitment and study design

**Fig. 1:**
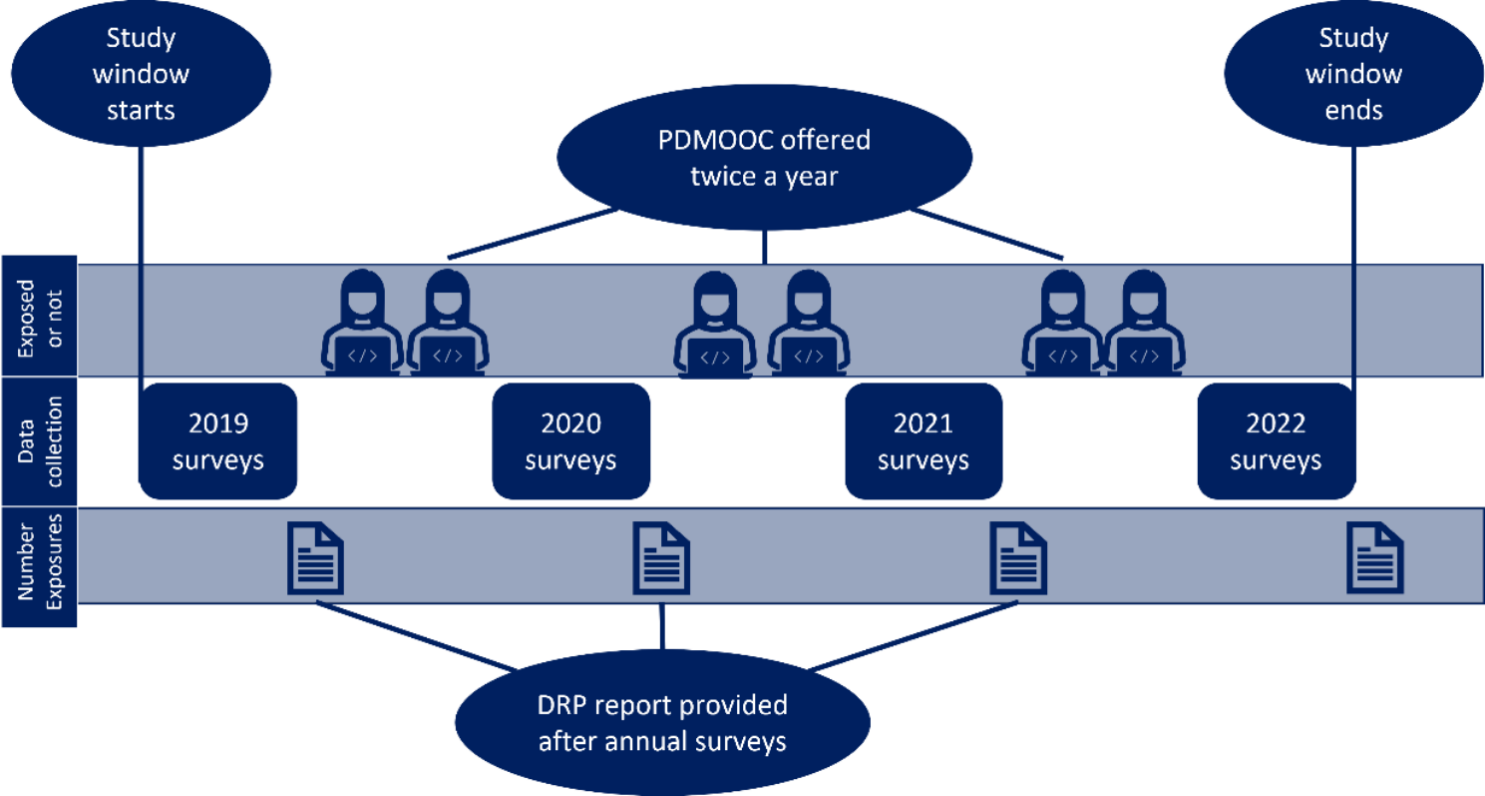
Study design diagram

ISLAND is a dementia prevention public health study with nested dementia risk reduction interventions [20]. Participants in this 10-year project were recruited via open invitation to become part of a large, prospective online dementia prevention initiative taking place in Tasmania, Australia. This invitation was widely disseminated via print, social and news media, posters, community information sessions and health events and was open to anyone with an internet connection and email address, aged 50 years or over and residing in Tasmania. These minimal eligibility criteria, and the online design of the study, were intended to maximise inclusiveness. Participants were asked to read the study information sheet and provide informed consent to the research conditions prior to baseline and all subsequent surveys. After consenting to join the research, participants completed a baseline survey battery, assessing their dementia risk knowledge, motivations to change dementia risk and behaviours related to nine modifiable dementia risk factors. Baseline could be completed at any time in the first three years of the project. Invitations to do the October annual surveys were extended to all participants whose baseline was completed no less than five months before each October. Annual invitations to do the follow-up surveys were then sent in October every year for the study duration. While the planned annual surveys for ISLAND will run until 2029, the study window for the current paper is illustrated in Fig. 1. Analyses were conducted using data from participants who had not engaged with the PDMOOC prior to joining ISLAND, with baseline data provided between October 2019 and June 2022, and at least one follow-up survey, with the latest being the October 2022 annual assessments. All surveys were administered online via a secure password-protected portal, allowing the linking of multisource data, and tracking of engagement with ISLAND interventions and assessments.

### Interventions

The printable DRP report reflects, in traffic light format, the information participants entered into their ISLAND surveys. Table 1 presents the cut points used for determining risk level for each assessed modifiable risk factor [6, 7, 26, 32]. The use of red for high-risk, orange for medium-risk and green for low-risk scores per risk domain is easily interpretable and the report includes recommendations for action customised to these risk scores. An individual’s DRP report might suggest: drinking less than two standard alcoholic drinks per day, not smoking, engaging regularly in 150 min per week of moderate physical activity, adhering to a Mediterranean style diet and keeping a healthy body-mass index (BMI), ensuring cardiometabolic conditions (hypertension, dyslipidaemia, diabetes) are medically managed, and engaging in social and cognitively stimulating activities. The DRP report was made available immediately after baseline and updated versions were released after participants completed each wave of the annual ISLAND surveys. Participants were encouraged to discuss their DRP report with their health care providers, and the user portal enabled participants to easily track year-on-year changes in their behavioural risk profile over time. More generic health promotion messages about how and why making small changes toward these health goals can support brain health are disseminated to ISLAND participants via email-based newsletters, and public broadcast via local radio, print newspapers and social media. Through these pathways, ISLAND participants were encouraged to enrol and take part in the PDMOOC, which provides the opportunity to engage in self-education about dementia risk reduction [29]. The PDMOOC is an established course ranked in the top health MOOCs worldwide [28]. Using formal, online educational methods the PDMOOC presents, discusses and reinforces evidence-based information on what modifiable dementia risk factors are, why they are important and what can be done to address them [29]. This 4-week online course conveys established and emerging evidence regarding modifiable dementia risks using print materials, videos, quizzes and discussion boards. Participants incur no course fees, except for a small administration charge if they elect to receive a PDMOOC completion certificate. The PDMOOC has been well received by online learners since 2016 [28] and has shown promise as a dementia risk reduction intervention [29].

### Measures

Established research questionnaires were used when available (e.g. motivations to change behaviours for dementia risk reduction [19, 33]), however novel questionnaires were applied to assess knowledge and behaviours specific to dementia risk reduction [26, 34]. The novel measures are being separately validated.

Demographic factors age, gender, educational attainment, marital and socioeconomic status are understood to be associated with physical and mental health [35]. These factors also influence dementia risk [5] and were therefore included as covariates in regression models. Socioeconomic status was assigned a decile (range 1:10) using postcode data and based on the Australian Bureau of Statistics’ Index of Relative Socio-economic Disadvantage (IRSAD) [36]. A low IRSAD score means the participant resides in an area where no formal qualifications, low skills-based employment and low income are common, whereas high scores mean high incomes, skills-based employment and academic qualifications are common.

Dementia risk behaviours were measured up to four times, at baseline and all subsequent data collection waves (Fig. 1) using responses to the questions that generated the personal DRP reports. Cut points for risk levels (low, medium and high) based on established guidelines [6, 7] (Table 1) were applied to compute each participant’s risk status for the assessed dementia risk behaviour domains. These categorical data were then used to understand the proportion of participants who changed risk status during the study period. Cumulative change across these nine risk factors was measured by summing scores across the domains, where low risk = ‘0’, medium risk = ‘0.5’ and high risk = ‘1’. This procedure provided a defensible continuous measure of dementia risk behaviours (DRP total, range 0 to 9), with lower scores indicating lower risk.

**Table 1:** Assignment of risk level for the assessed modifiable risk factors

Risk domain	Risk level	Cut points
Alcohol consumption		
	Low	Not drinking alcohol, or no more than 2 standard drinks per occasion
	Medium	14 or less standard drinks per week and either more than 2 standard drinks per occasion
	High	More than 14 standard drinks per week
Blood pressure, cholesterol and diabetes management		
	Low	No diagnosis and regular check-ups; or diagnosis and medically managed
	Medium	Unsure of diagnosis but have regular check-ups; or diagnosis with regular check-ups and working toward managing it
	High	Diagnosis but no regular check-ups and/or medical management; or no diagnosis and no regular check-ups
Body mass index (weight in kgs/height in metres)		
	Low	18 to 24.9
	Medium	25 to 29.9; or less than 18
	High	30 or over
Adherence to the MIND diet (score range 0:14)		
	Low	12 or higher
	Medium	7.5 to 11.9
	High	Less than 7.5
Smoking		
	Low	Not smoking
	Medium	Occasional smoking
	High	Smoking once or more a week
Physical activity (METs)		
	Low	600 or more
	High	Less than 600
Cognitive activity (frequency of 11 cultural and cognitive activities)		
	Low	33 or higher
	High	Less than 33

MIND: Mediterranean-DASH Intervention for Neurodegenerative Delay diet (excluding wine consumption) [37] was scored by summing the 15 component scores. Physical activity assessed based on METS: metabolic equivalent of tasks, where light, moderate and vigorous activity minutes were assigned scores of 3.3, 4 and 8 respectively; 600 MET = sufficient (low risk) if ≥ 150 min of moderate activity per week. Cognitive activity items were adapted from the ANU-ADRI (32); with sufficient (low risk) indicated when ‘several times a month’ (3) was selected for 11 cognitively stimulating activities.

The novel Knowledge of Dementia Risk Reduction (KoDeRR) survey [34] was used to assess changes dementia risk knowledge over time and by intervention exposure. This instrument provides scores for three aspects of knowledge: general knowledge of dementia risk (range 0 to 22), knowledge of evidence-based strategies to reduce modifiable risk factors (range 0 to 20) and the correct identification of misconceptions about strategies to mitigate risk factors (range 0 to 12). Higher scores for each component of the KoDeRR indicate higher levels of dementia risk knowledge. The misconceptions element of the KoDeRR survey was selected for the planned mediation analyses because it provides an indication of discernment, or the ability to determine the accuracy and applicability of available information, and is thus a stronger indicator of actionable knowledge than general knowledge alone [17, 25].

Motivations and attitudes about changing lifestyle and health behaviours to reduce dementia risk were assessed using a measure developed by Kim, Sargent-Cox (19; MCHLB-DRR). This 28-item instrument includes seven dimensions that align with the HBM approach to explaining behaviour change [17]: perceived susceptibility (range 5 to 20), severity (range 6 to 25), benefits (range 5 to 20) and barriers (range 5 to 20) to changing a behaviour, cues to action (range 5 to 20), self-efficacy (range 2 to 10) and general health motivations (range 5 to 20). Higher scores on the two threat-oriented dimensions (perceived susceptibility and perceived severity) are thought to motivate action-taking via a fear of developing dementia. Perceived benefits of changing behaviours, cues to action, self-efficacy and general health motivations are resource-based motivators. Higher scores on these dimensions of the MCHLB-DRR indicate stronger motivation to change risk behaviours. In contrast, when perceived barriers to taking action are high, motivation to change risk behaviours is inhibited. Perceived susceptibility and self-efficacy were selected from the full instrument for the planned mediation analyses, to examine the influence on the knowledge-to-behaviour-change path of threat-based and resource-based motivational factors.

### Analytic strategy

Analyses were conducted in the R environment for statistical computing [38]. Two-sided tests of statistical significance used α = 0.05. We report descriptive means in tables and estimated marginal means, standardized coefficients (*b*_*s*_) and odds-ratios (OR) with 95% confidence intervals to indicate effect size and certainty for regression and mediation results. We adjusted α to control family-wise error rate using the Tukey method (detailed below) but did not otherwise adjust α or standard errors to control false discovery rate. Cases were excluded from analyses when data for individual domains within the DRP were coded as ‘unknown’. To accommodate the rolling baseline design and ensure effects were assessed over comparable time periods, time (months) in ISLAND was computed for each participant. As illustrated in Fig. 1, exposure to the DRP occurs after completing each survey wave. This means participants who provided baseline and just the 2022 follow-up survey would have had one exposure to the DRP within the study window and two waves of data. Those with data provided at baseline in 2019, and in the annual surveys in 2020, 2021 and 2022 would have three DRP exposures and four waves of data. Exposure to PDMOOC was coded as a dummy variable, with ‘0’ indicating no exposure and ‘1’ indicating they had completed at least the first of four modules. This cut point was based on records showing most participants who complete the first module go on to finish the course [29]. Participants who had enrolled in the PDMOOC prior to joining ISLAND (and therefore had a record on the learning management system) were assigned ‘1’ at baseline, to avoid confounding in analyses of intervention effects.

Aim 1: The primary outcome for this study was the cumulative number of medium and high risk DRP scores (DRP total). The explanatory variables were knowledge about and motivations to change dementia risk behaviours. Exposures were time, number of DRP exposures and engagement (or not) with the PDMOOC.

Time in ISLAND, calculated as the number of months since first exposure to the DRP (immediately after BL), showed a nonlinear pattern and was transformed (log months + 1) prior to entry into the regression models. The DRP total score and survey data from the knowledge and motivations surveys were continuous variables and observed changes were estimated with univariate linear mixed effects regression models fitted by restricted maximum likelihood (REML). Omnibus changes in the primary (behaviours) and explanatory (knowledge and motivations) variables were assessed first (H1), followed by the effects on these variables of the PDMOOC (H2). Post-hoc contrasts were applied using the Tukey method for multiple comparisons. To obtain odds-ratios for changes observed over time in each domain of the dementia risk profile, multinomial logistic regression was applied to the categorical DRP data.

Aim 2: To test the influence of knowledge and motivational factors on change in DRP total, three mediation analyses were conducted using the lavaan package [39]. First the influence of knowledge on the path between PDMOOC exposure and behaviour change (H3) was tested; then two dimensions of the motivations instrument were tested for their mediating influence on the path between increased knowledge and behaviour change (H4 and H5). Linear regression was used to estimate coefficients, adjusting each outcome (observations recorded in October 2022) for its corresponding observation at baseline. The indirect path was estimated using the product of coefficients method. We evaluated model fit using root mean square error of approximation (RMSEA), and comparative fit index (CFI). Parameters were estimated using full information maximum likelihood (FIML) to account for missing observations. Standard errors were bootstrapped using 5000 replications. Reproducible R code is provided in Additional File 1.

## Results

### Participation and attrition

Participant demographics are reported in Table 2. The total ISLAND baseline sample (n = 7264) had a mean age of 63.2 (SD = 7.85) years, was predominantly female (71.2%), married (70.4%) and university qualified (50.4%). The analysis sample (n = 3038) comprised 41.8% of the total ISLAND research sample. Over half (n = 2017, 58.8%) engaged with the PDMOOC during the current study window. Compared to those not included in the reported analyses, baseline data showed the analysis sample was older (63.7 vs. 62.9 years) and reported fewer risk factors (2.17 vs. 2.34), higher rates of university-level qualification (55.3% vs. 46.9%), retirement (51.5% vs. 44.8%), and socioeconomic advantage (29.3% vs. 25.7%).

**Table 2:** Baseline characteristics of the analysis sample relative to the ISLAND starting sample

Characteristics at baseline	Entire ISLAND sample (N = 7264)		Not included (n = 4226)		Analysis sample (n = 3038)		P-value	
**Age (whole sample)**								
Mean (SD)	63.2	(7.85)	62.9	793.00	63.7	7.73	< 0.001	
Median [Min, Max]	63	[50, 94]	62	[50, 94]	63	[50, 91]		
**Age categories (n, %)**								
50–59	2576	35.5	1601	37.90	975	32.10	< 0.001	
60–69	3071	42.3	1720	40.70	1351	44.50		
70–79	1420	19.5	782	18.50	638	21.00		
80+	197	2.7	123	2.90	74	2.40		
**Marital status (n, %)**								
Married/Defacto	5112	70.4	2913	68.90	2199	72.40	0.002	
Not Married/Defacto	2152	29.6	1313	31.10	839	27.60		
**Gender (n, %)**								
Female	5171	71.2	2995	70.90	2176	71.60	0.750	
Male	2082	28.7	1225	29.00	857	28.20		
Other	11	0.2	6	0.10	5	0.20		
**Employment status (n, %)**								
Employed	3947	54.3	2177	51.50	1770	58.30	< 0.001	
Retired	3456	47.6	1892	44.80	1564	51.50	0.011	
**Educational attainment (n, %)**								
University qualification	3663	50.4	1983	46.90	1680	55.30	< 0.001	
Post-secondary	2188	30.1	1339	31.70	849	27.90		
School only	1136	15.6	742	17.60	394	13.00		
Unknown	277	3.8	162	3.80	115	3.80		
**Socioeconomic advantage (n, %)**								
Advantaged	1974	27.2	1085	25.70	889	29.30	< 0.001	
Mid-range	2668	36.7	1547	36.60	1121	36.90		
Disadvantaged	2574	35.4	1571	37.20	1003	33.00		
**Residential remoteness (n, %)**								
Inner regional	5265	72.5	3036	71.80	2229	73.40	0.043	
Outer regional	1904	26.2	1130	26.70	774	25.50		
Remote	28	0.4	19	0.40	9	0.30		
Very remote	26	0.4	21	0.50	5	0.20		
**Number of high risk factors**								
Mean (SD)	2.27	(1.26)	2.34	128.00	2.17	1.24	< 0.001	
Median [Min, Max]	2	[0, 8.5]	2	[0, 8.5]	2	[0, 8.0]		

Baseline characteristics of the full ISLAND sample, those not included and the analysis sample used for this paper. Test of difference = p-value from t-test or χ^2^ test of difference (with α = 0.05) between the analysis sample and participants not included (i.e. who did the PDMOOC prior to joining ISLAND or did not respond to the 2022 annual surveys).

### Dementia risk behaviours

Table 3 presents the proportion of participants by risk level and timepoint for each of the assessed dementia risk behaviours, and the DRP total score. Sample-wide results show a small reduction in mean DRP total scores for each exposure to the DRP report (*b*_*s*_ = − 0.03 [95%CI − 0.07, − 0.00]), adjusted for the number of months in the study (p < .001). Estimated marginal means per timepoint from the omnibus REML analyses are presented in Supplementary Table S1 (Additional File 1).

**Table 3:** Descriptive summary data showing risk levels by timepoint for participant-reported dementia risk behaviours and lifestyle factors

Dementia risk factor	Baseline	Oct-20	Oct-21	Oct-22
	n = 3038	n = 1995	n = 2099	n = 2992
Total Score				
Mean (SD)	2.17 (1.24)	1.74 (1.13)	1.61 (1.12)	1.66 (1.11)
Median [Min, Max]	2.00 [0, 8.00]	1.50 [0, 6.50]	1.50 [0, 6.50]	1.50 [0, 6.50]
Alcohol Risk				
high	390 (12.8%)	175 (8.8%)	173 (8.2%)	258 (8.6%)
medium	909 (29.9%)	548 (27.5%)	525 (25.0%)	773 (25.8%)
low	1533 (50.5%)	1201 (60.2%)	1307 (62.3%)	1806 (60.4%)
unknown	206 (6.8%)	71 (3.6%)	94 (4.5%)	155 (5.2%)
BMI Risk				
high	663 (21.8%)	403 (20.2%)	438 (20.9%)	657 (22.0%)
medium	1037 (34.1%)	716 (35.9%)	731 (34.8%)	1089 (36.4%)
low	1204 (39.6%)	871 (43.7%)	928 (44.2%)	1240 (41.4%)
unknown	134 (4.4%)	5 (0.3%)	2 (0.1%)	6 (0.2%)
Hypertension Risk				
high	123 (4.0%)	84 (4.2%)	76 (3.6%)	116 (3.9%)
medium	69 (2.3%)	40 (2.0%)	55 (2.6%)	74 (2.5%)
low	2831 (93.2%)	1865 (93.5%)	1965 (93.6%)	2798 (93.5%)
unknown	15 (0.5%)	6 (0.3%)	3 (0.1%)	4 (0.1%)
Cholesterol Risk				
high	438 (14.4%)	228 (11.4%)	221 (10.5%)	287 (9.6%)
medium	110 (3.6%)	96 (4.8%)	71 (3.4%)	151 (5.0%)
low	2472 (81.4%)	1666 (83.5%)	1803 (85.9%)	2553 (85.3%)
unknown	18 (0.6%)	5 (0.3%)	4 (0.2%)	1 (0.0%)
Diabetes Risk				
high	482 (15.9%)	249 (12.5%)	234 (11.1%)	324 (10.8%)
medium	41 (1.3%)	28 (1.4%)	30 (1.4%)	42 (1.4%)
low	2490 (82.0%)	1716 (86.0%)	1835 (87.4%)	2624 (87.7%)
unknown	25 (0.8%)	2 (0.1%)	0 (0%)	2 (0.1%)
Smoking Risk				
high	59 (1.9%)	29 (1.5%)	33 (1.6%)	49 (1.6%)
medium	41 (1.3%)	12 (0.6%)	14 (0.7%)	27 (0.9%)
low	2932 (96.5%)	1945 (97.5%)	2051 (97.7%)	2913 (97.4%)
unknown	6 (0.2%)	9 (0.5%)	1 (0.0%)	3 (0.1%)
Cognitive Activity Risk				
high	1242 (40.9%)	567 (28.4%)	440 (21.0%)	622 (20.8%)
low	1763 (58.0%)	1385 (69.4%)	1600 (76.2%)	2307 (77.1%)
unknown	33 (1.1%)	43 (2.2%)	59 (2.8%)	63 (2.1%)
Physical Activity Risk				
high	269 (8.9%)	82 (4.1%)	92 (4.4%)	159 (5.3%)
low	2677 (88.1%)	1902 (95.3%)	1998 (95.2%)	2818 (94.2%)
unknown	92 (3.0%)	11 (0.6%)	9 (0.4%)	15 (0.5%)
Mediterranean Diet Risk				
high	171 (5.6%)	79 (4.0%)	81 (3.9%)	138 (4.6%)
medium	2220 (73.1%)	1344 (67.4%)	1363 (64.9%)	2008 (67.1%)
low	633 (20.8%)	548 (27.5%)	640 (30.5%)	830 (27.7%)
unknown	14 (0.5%)	24 (1.2%)	15 (0.7%)	16 (0.5%)

BL: baseline completed between Oct 2019 and June 2022; Oct-20: data from sample with BL prior to August 2020 and 2020 survey data; Oct-21: data from sample with BL prior to August 2021 and 2021 survey data; Oct-22: data from sample with BL prior to August 2022 and survey data in 2022.

**Fig. 2:**
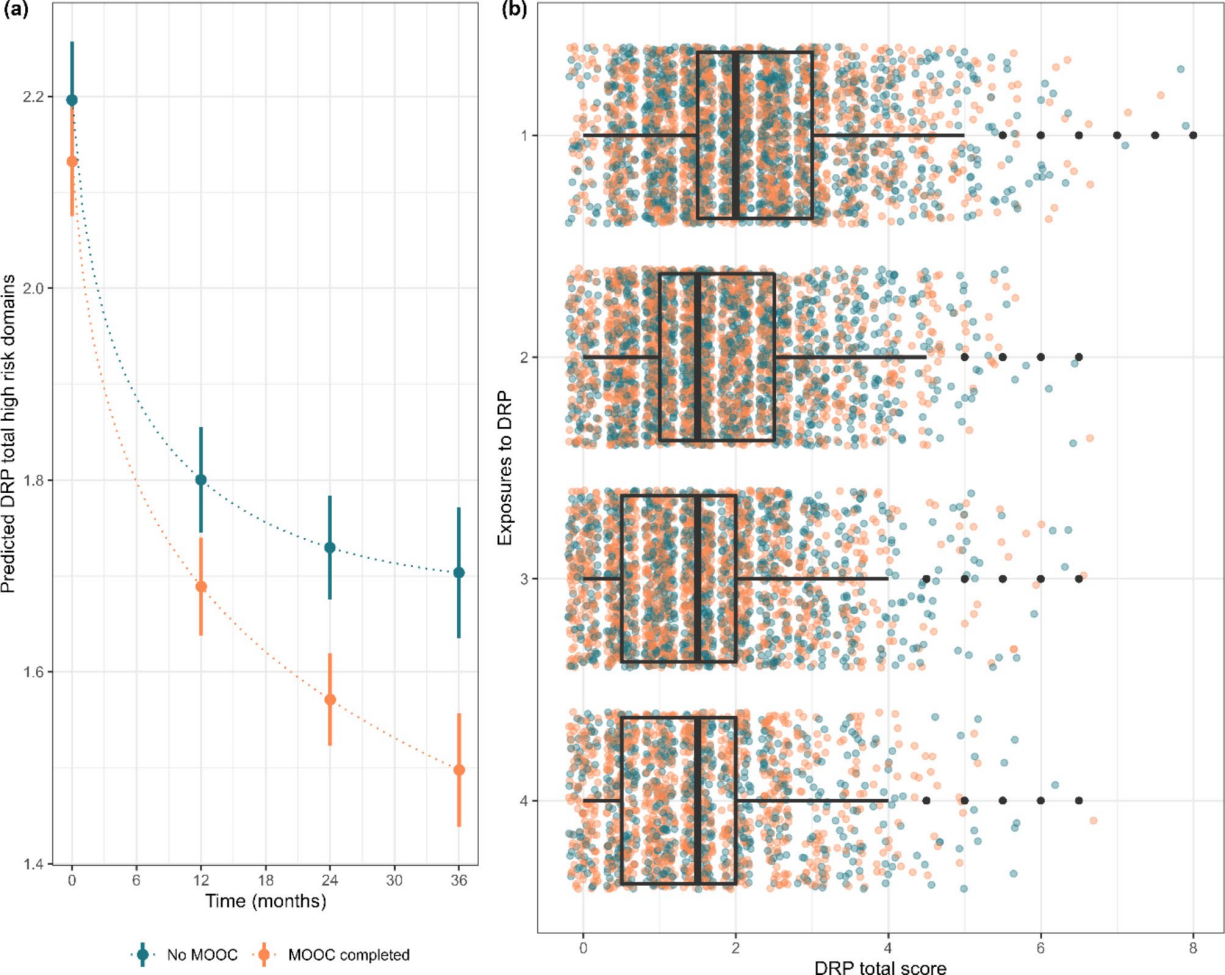
Change over time in ISLAND participants’ dementia risk profile (DRP) score by engagement with the Preventing Dementia Massive Open Online Course (PDMOOC) (n = 3038). (a) shows mean scores (dots) and 95% confidence intervals (vertical lines) by time and (b) shows the distribution of individual cases by exposure to the personalised Dementia Risk Profile report, Box and whisker plots overlaid on individual data points illustrate where the mean and 95% confidence intervals lie)

Results for the tests of difference in DRP total by PDMOOC engagement are illustrated in Fig. 2a and b with estimated marginal means presented in Supplementary Table S2 (Additional File 1). Visual inspection of Fig. 2a reveals that while a decline continued for subsequent exposures, the strongest reduction in DRP total occurred after the first exposure to the personal DRP report. Figure 2b shows the distribution of individual DRP total scores by PDMOOC exposure at baseline and subsequent surveys. PDMOOC engagement was associated with a stronger reduction in DRP total scores than was observed in non-participants (*b*_*s*_ = − 0.10 [95%CI − 0.16, − 0.04], p = .001). There was a significant interaction effect on risk behaviours of the number of DRP exposures and PDMOOC engagement (*b*_*s*_=-0.03 [95%CI − 0.06, 0.00]). This result was retained when demographic covariates were included, and the amount of variance accounted for increased from Marginal R^2^ = 0.044 to R^2^ = 0.098.

The sample-wide probability of transitioning into and out of risk levels from baseline to 2022 was estimated for each of the nine domains of risk behaviour, adjusted for gender, age, socioeconomic status and education (Supplementary Table S3, Additional File 1). Results show a significant proportion of participants who reported high or medium-risk status at baseline shifted into a lower risk status for almost all assessed behaviours. Body mass index (BMI) was the only risk domain where the proportion of the sample shifting from high-risk to medium, or from medium-risk to low-risk status did not achieve significance. We conducted contrast multinomial regression analyses to understand the expected effects of engaging with the PDMOOC (vs. not) on individual DRP domains (Supplementary Table S4, Additional File 1). PDMOOC engagement significantly increased the odds of participants shifting out of the high- and medium-risk and into low-risk category for diabetes management (5%) and adherence to a Mediterranean style diet (4%). Following PDMOOC engagement, participants were less likely to be in the medium risk category for smoking (5%), however changes in smoking rates by PDMOOC engagement were not consistently in the beneficial direction. No other significant effects of the PDMOOC were observed at domain level.

### Dementia risk knowledge

Descriptive data from the KoDeRR instrument for each timepoint are in Supplementary Table S5 (Additional File 1). Omnibus regression results show general knowledge of dementia risk increased across the sample with the number of DRP exposures (*b*_*s*_=0.13 [95%CI 0.12, 0.14]). There was a higher rate of correct attributions of modifiable risk factors (*b*_*s*_=0.44 [95%CI.43, 0.45]) and discernment of misconceptions (*b*_*s*_=0.11 [95%CI 0.10, 0.12] in follow-up data compared with baseline. PDMOOC engagement was consistently associated with stronger change in risk knowledge than the DRP alone: general knowledge (*b*_*s*_=0.26 [95%CI.20, 0.32]), modifiable risk factor identification (*b*_*s*_=0.13 [95%CI 0.08, 0.19]) and misconception discernment (*b*_*s*_=0.35 [95%CI 0.29, 0.41]). The interaction of DRP exposures and PDMOOC engagement showed that participants who undertook the course reported stronger change in general knowledge (*b*_*s*_=0.07 [95%CI 0.04, 0.09]), risk identification (*b*_*s*_=0.07 [95%CI 0.04, 0.09]) and misconception discernment (*b*_*s*_=0.15 [95%CI 0.12, 0.17]) when compared with participants who did not do the PDMOOC.

### Motivations to reduce dementia risk

Results from the models testing the influence of ISLAND interventions on attitudinal and motivational factors [19] showed the number of exposures to the DRP was associated with a significant, small reduction in perceived susceptibility to dementia (*b*_*s*_=-0.04 [95%CI − 0.05, − 0.03]), and perceived severity of a dementia diagnosis (*b*_*s*=_-0.04 [95%CI − 0.05, − 0.02]). Similarly small increases were noted for perceived benefits of action-taking (*b*_*s*_=0.03 [95%CI 0.02, 0.04]) and cues to action (*b*_*s*_=0.02 [95%CI.00, 0.03]). The effect of DRP exposures on perceived barriers to action taking (*b*_*s*_=0.03 [95%CI 0.01, 0.04]) and general health motivations (*b*_*s*_ = − 0.02 [95%CI − 0.04, − 0.01]) both showed a small detrimental change, and self-efficacy did not significantly change by time and DRP exposure alone.

Comparing PDMOOC participants with non-participants, significant improvements were observed for self-efficacy (*b*_*s*_=0.15 [95%CI 0.10, 0.21]), perceived benefits (*b*_*s*_=0.11 [95%CI 0.05, 0.17]), perceived barriers (*b*_*s*_=-0.19 [95%CI − 0.25, − 0.13]), and a smaller effect for general health motivations (*b*_*s*_=0.08 [95%CI 0.02, 0.14]). The interaction between DRP exposures and PDMOOC engagement showed that, compared with participants who did not do the PDMOOC, those who did the course reported a stronger reduction in perceived susceptibility to dementia (*b*_*s*_=-0.04 [95%CI − 0.07, − 0.02]), lower barriers to action (*b*_*s*_=-0.04 [95%CI − 0.06, − 0.01]) and higher self-efficacy (*b*_*s*_=0.04 [95%CI 0.01, 0.07]) with DRP exposure. No significant interaction effect of the combined interventions was observed for perceived severity, perceived benefits, cues to action or general health motivations.

### Dementia risk knowledge and motivations as mediators of change in risk behaviours

**Fig. 3:**
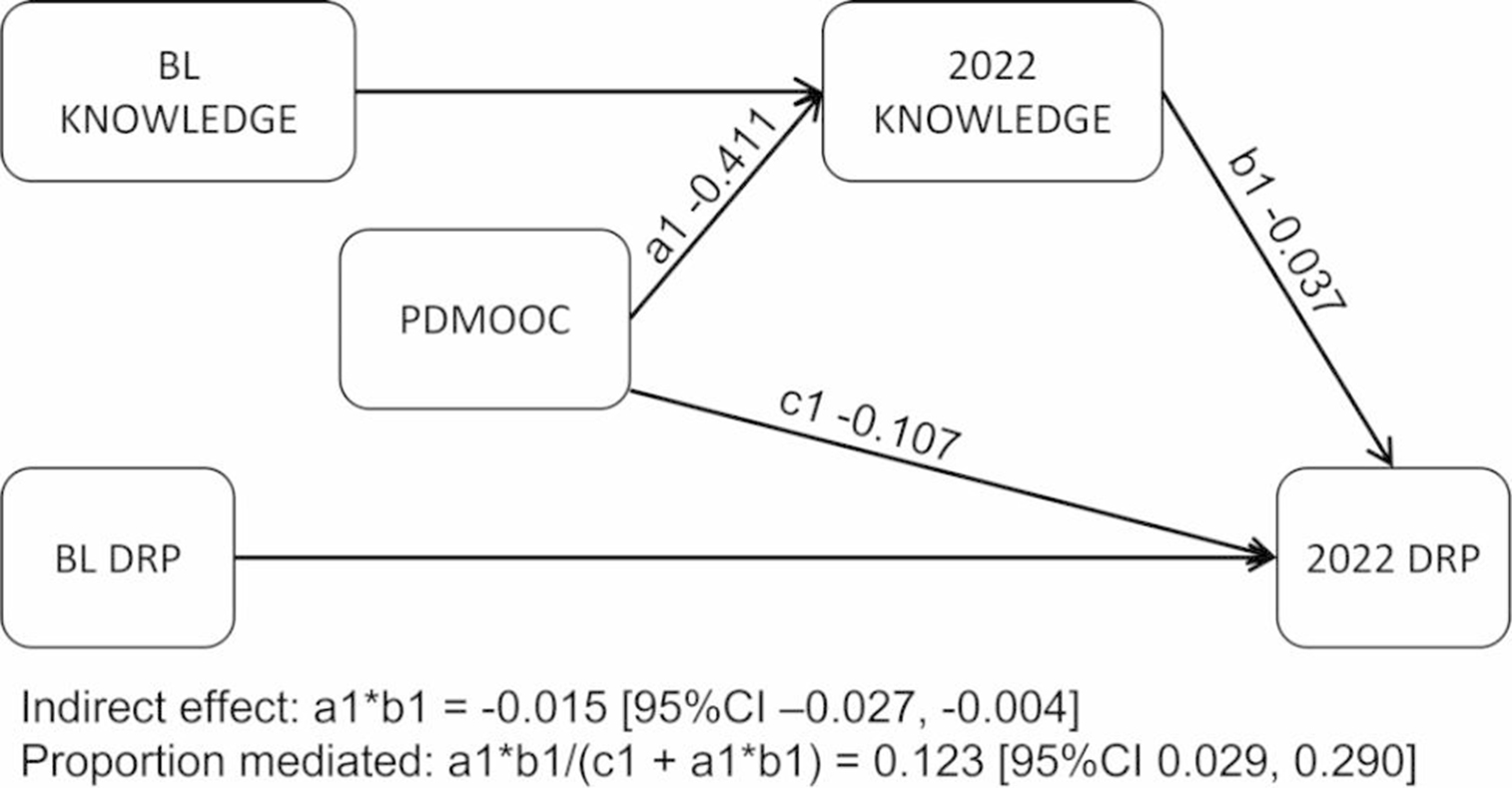
Mediating role of misconception identification on the effect of the PDMOOC on dementia risk behaviours (n = 3038). Full information maximum likelihood structural equation model with bootstrapping set to 5000. BL: baseline; Knowledge: correct identification of misconceptions about dementia risk; DRP: Dementia risk profile total score; PDMOOC: Preventing Dementia Massive Open Online Course

Figure 3 shows the mediating role of knowledge on the path between intervention and behaviour change. This model used data from 3038 participants and had good fit indices (CFI = 0.995, RMSEA = 0.043 [90%CI 0.023, 0.066]). Compared with non-PDMOOC participants, participation in the PDMOOC was directly associated with reduced DRP scores (-0.11 SD [95%CI − 0.17, − 0.05], p < .001), and an increase in knowledge (0.41 SD [95% CI 0.35, 0.47], p < .001). Our mediation results showed changes in knowledge mediated 12% (SE 0.07) of the beneficial effect of the ISLAND interventions (including the PDMOOC) on the number of reported risk factors (DRP total) (-0.02 SD [95%CI − 0.03, − 0.00], p = .013).

**Fig. 4:**
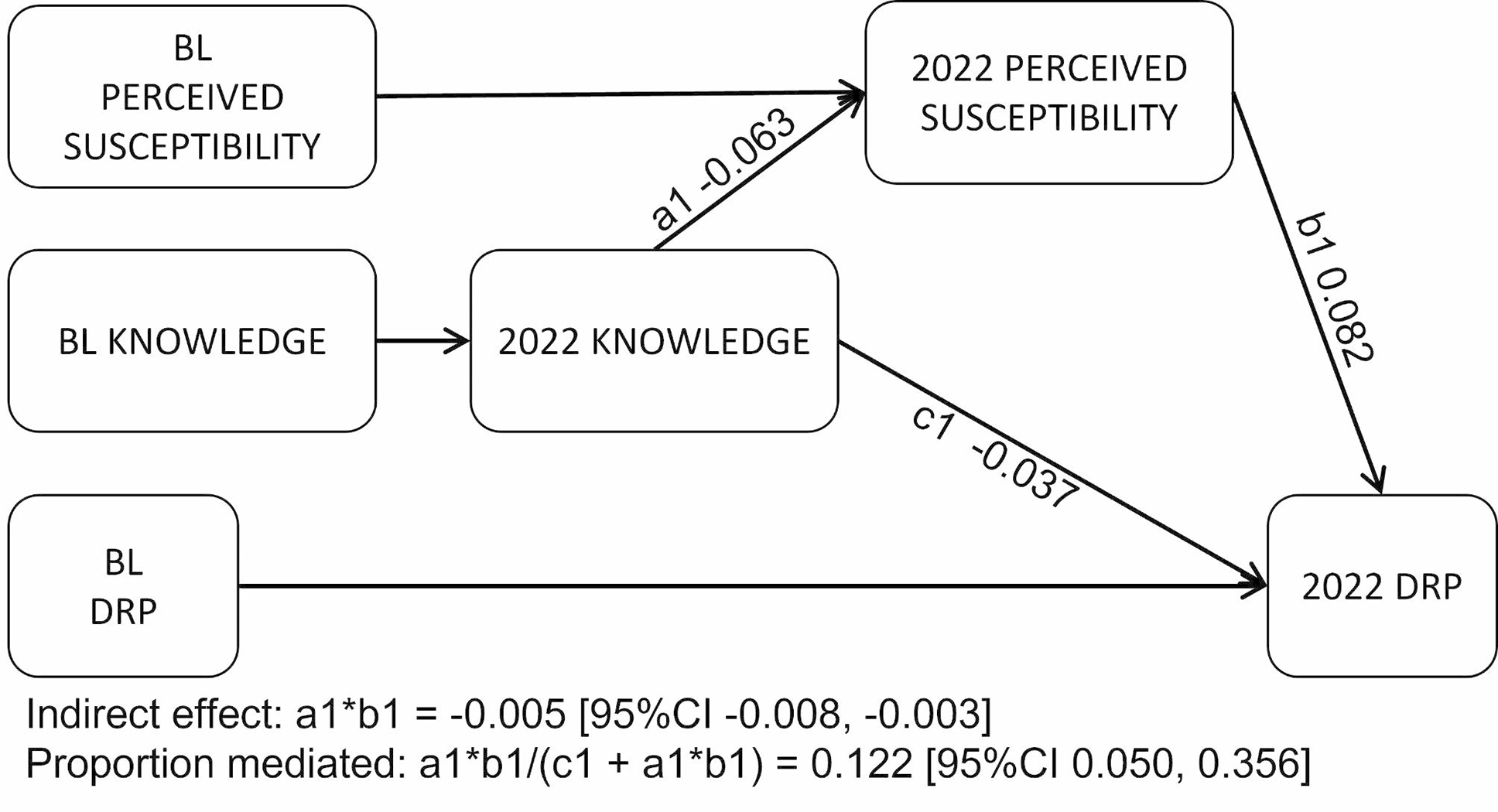
Mediating role of perceived susceptibility to dementia on the path between knowledge on risk behaviours (n = 3001). Full information maximum likelihood structural equation model with bootstrapping set to 5000. BL: baseline; Knowledge: correct identification of misconceptions about dementia risk; DRP: Dementia risk profile total score; PDMOOC: Preventing Dementia Massive Open Online Course

Figure 4 is a path diagram showing results for the perceived susceptibility model. Data from 3001 participants were used and fit indices were good (CFI = 0.996, RMSEA = 0.045 [90%CI 0.025, 0.069]). Results showed higher perceived susceptibility to dementia was associated with a higher number of risk behaviours (0.08 SD [95%CI 0.05, 0.11], p < .001). Perceived susceptibility to dementia had a small attenuating effect on the positive effect of higher knowledge on behaviours, as it mediated 12% (SE 0.38) of the influence of knowledge on DRP scores (-0.01 SD [95%CI − 0.01, − 0.00] p = .001).

**Fig. 5:**
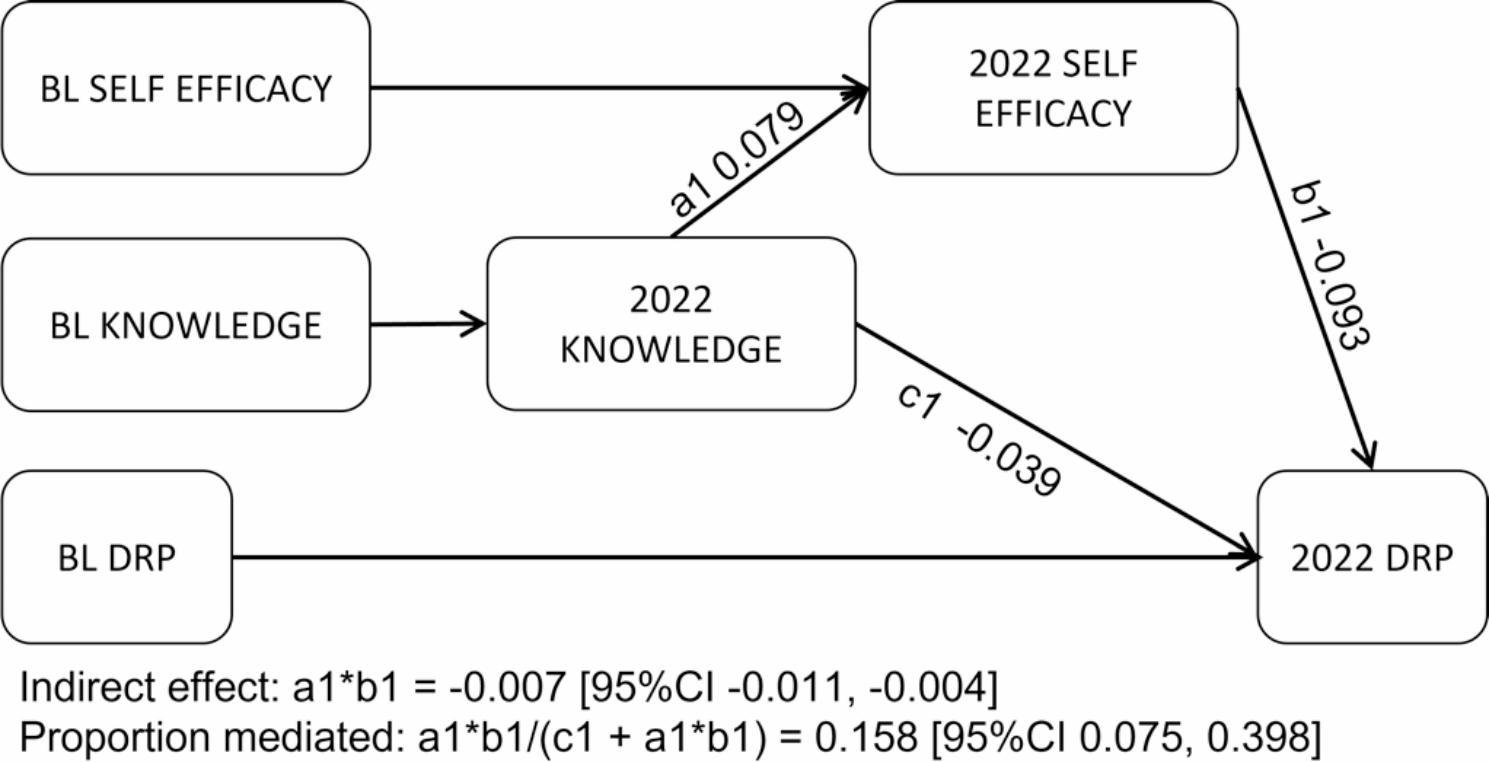
Mediating role of self-efficacy on the path between knowledge and risk behaviours (n = 3001). Full information maximum likelihood structural equation model with bootstrapping set to 5000. BL: baseline; Knowledge: correct identification of misconceptions about dementia risk; DRP: Dementia risk profile score; PDMOOC: Preventing Dementia Massive Open Online Course

The third model in the mediation series tested the influence of self-efficacy on the path between increased knowledge and fewer dementia risk behaviours (Fig. 5). Model fit for the included 3001 observations was good (CFI = 0.990, RMSEA = 0.052 [90%CI 0.032, 0.075]). Results showed higher self-efficacy was associated with lower DRP total scores (-0.09 SD [95%CI − 0.12, − 0.06] p < .001) and mediated 16% (SE 0.205) of the influence of increased knowledge on risk behaviour change (-0.01 SD [95%CI − 0.01, − 0.00] p < .001).

## Discussion

This paper presents patterns of change observed in knowledge, motivations and behaviours related to modifiable dementia risk factors over the first three years (2019 to 2022) of the ISLAND project [20]. We investigated theoretically supported aspects of dementia risk knowledge and motivations to change behaviours as process variables, to help explain the observed effects of the ISLAND interventions on dementia risk behaviours. We found providing personalised and actionable feedback via the DRP report and the option to learn more about dementia risk reduction via the PDMOOC can help support people to better manage their own health risk behaviours. These results indicate ISLAND offers an intervention framework for translating health promotion and behaviour change research into practice [11].

Within three years of commencement, the number of modifiable dementia risk factors in a sample of 3038 Tasmanian residents reduced from 2.16 out of 9 risk domains at baseline, to 1.66 in 2022. Even considering the healthy, educated and relatively advantaged nature of the ISLAND cohort, this equates to a 26% improvement in risk factor status over three years. Ashby-Mitchell et al. [40] projected a 5%, 10%, 15% and 20% change in each risk factor would reduce dementia prevalence by between 3.3% and 14.9% over a 20 year period. While it is not feasible to directly apply Ashby-Mitchell’s model because we have assessed multiple co-occurring risk domains, the changes observed in ISLAND participants have the potential, if retained over the long term, to help reduce the future dementia prevalence for the participating sample.

Exposure to the dementia risk profile (DRP) report was associated with reduced alcohol consumption, improved adherence to the Mediterranean style diet, increased physical activity, quitting smoking, and improved management of blood pressure, diabetes and cholesterol. These results suggest the personalised DRP report is a helpful public health intervention for supporting dementia prevention [5, 6]. Because of the number of risk factors that dementia has in common with cardiometabolic diseases, the DRP may also be a useful support for preventing or better managing other age- and lifestyle-related health conditions [41].

Premised on prior evidence showing health beliefs and attitudes towards dementia influence behaviour change intentions [22], our first hypothesis (H1) that knowledge, motivations and behaviours related to modifiable dementia risk would improve over time for participants in the ISLAND project was supported. The results suggest providing customized feedback and guidance through the DRP report as an early and repeated part of engagement with the project enables ISLAND participants not just to learn what is known about modifiable dementia risk factors, but to find out which risk factors they have and what they can do about them. While the feasibility of providing personalised risk profiles was supported in a separate proof of concept trial [14], our results provide new and compelling evidence that this approach is not just feasible but also effective when implemented at scale. However, alongside the benefits, exposure to the DRP was also associated with increased perceived barriers to changing behaviours and lower general health motivations, and was not independently associated with self-efficacy. While there was evidence of improvement in specific risk factors by DRP exposures, some changed more than others. It is possible that while the personalised DRP report helps develop the rationale and intentions for behaviour change, it may not – on its own – serve to increase personal resources that may be required for implementing those intentions. It is likely that achieving the recommended amount of physical exercise, improving diet, not smoking or drinking to excess and making a doctor’s appointment to monitor cardiometabolic health indices are conceptually and practically easier to implement than a more general instruction to lose weight, which is understood to be difficult for many people [42].

Our second hypothesis (H2), that engagement with a four-week online course (the PDMOOC) would strengthen the effects of ISLAND on knowledge, motivations and behaviours related to dementia risk, was also supported. The high level of uptake (58.8% engagement) suggests the PDMOOC has broad appeal – which is as important to consider as efficacy – when studying the utility of public health and health promotion interventions [11].

It is possible the observed effects of the PDMOOC were due to the people who chose to do it rather than the course itself. Given the ISLAND sample consisted of people who had signed up to be involved in a dementia prevention study, the appeal of the PDMOOC is likely to have been stronger than would be observed in a general population sample. Further, PDMOOC participants had better baseline DRP scores and reported stronger effects in behaviour change over time than those who did not do the course. It is also acknowledged there were unmeasured factors at play (e.g. COVID-19, access to general practitioners, psychosocial resources) that are likely to have influenced the assessed health related behaviours and lifestyle factors over the study period. Despite these considerations, the significant interaction between the PDMOOC and DRP exposures was retained in the adjusted model, even though male gender, school-only education and living in a socioeconomically disadvantaged area were significant covariates.

While the results supported H2, it is worth noting the motivation dimensions affected by the PDMOOC are not the same as those affected by DRP exposures. Also, DRP exposures and PDMOOC engagement yielded a significant interaction effect on changes in risk behaviours. We propose the two interventions act synergistically, as they both target higher knowledge and personal motivations, albeit in different ways. It may be that while the DRP supports awareness of ones’ own risk profile and provides a stimulus to act, the PDMOOC offers guidance on why changing risk status is important and how to do so. The DRP and PDMOOC can therefore be seen as working together to increase knowledge about dementia risk and awareness of how this applies to oneself, thus stimulating the intention and the volition (self-efficacy) for behaviour change.

The mediation results support this interpretation and the central knowledge-to-behaviour-change hypothesis of ISLAND. Findings revealed that gains in dementia risk knowledge following the PDMOOC, specifically the correct identification of misconceptions, explained 12% of DRP improvement. Further, motivating factors – perceived susceptibility to dementia and self-efficacy for taking action to reduce dementia risk – were found to mediate 12% and 16% (respectively) of the influence of knowledge on risk behaviour change. Importantly, perceived susceptibility attenuated the beneficial impact of increased knowledge on risk behaviours, while self-efficacy was instrumental in supporting stronger changes in risk behaviours. Our mediation hypotheses (H3, H4 and H5), that changes in knowledge and motivations would mediate changes observed in risk behaviours following participation in the PDMOOC were supported. The results suggest different motivating factors have differential influences on the path between gaining actionable knowledge and behaviour change. It appears people who actively engage in formal dementia risk reduction education through the PDMOOC are likely to feel better equipped to take action, but also may find their beliefs about their susceptibility to disease can ‘get in the way’ of making changes to improve their risk profile.

### Limitations and future research

We acknowledge the ISLAND sample is disproportionately female, generally healthy, well-educated and relatively advantaged in socioeconomic terms, and therefore not truly representative of the Tasmanian population [20]. This non-representativeness is common in self-selecting samples, due to what is known as the ‘healthy volunteer’ effect, and is compounded by non-response bias in the sample used for analyses [44]. Further, while intended to overcome geographical access issues, the use of online methods required a level of literacy and access to technological infrastructure that likely presented barriers to participation. Thus, higher engagement by people at greater risk of dementia (i.e. with lower education, socioeconomic status, and poorer health risk profiles) might be better achieved if the interventions were tailored for delivery in community settings where social networks can be activated to support behaviour change. Our sample did not, on the whole, have high modifiable dementia risk scores at baseline, so there was limited room for sample-wide improvement. Also, the participants who engaged with the PDMOOC had better baseline risk profiles than those who did not do the course. Our results showing the effects of the PDMOOC are therefore subject to non-response bias. Results reported in the current paper do not provide evidence of differential changes based on the participants’ demographic characteristics. Future analyses are planned, that will show which risk behaviours tend to co-occur, with a view to tailoring intervention approaches that might best fit different risk behaviour and demographic phenotypes.

The influence of the COVID-19 pandemic, which emerged, during the study period, cannot be ignored, but was not expressly examined in this paper other than as an unmeasured potential modifying factor. Data that can be used to assess the effects and experiences of public health containment measures, virus exposures and other factors related to the pandemic will be investigated separately. This work will build on our previous analysis of changes in risk behaviours during lockdowns implemented between October 2019 and April 2020 [45], and changes in psychosocial dementia risk factors (social isolation, stress and depression), which were not included in the current paper. Importantly, the reported results do show a general improvement in the modifiable dementia risk profile of ISLAND participants despite the concurrent COVID-19 related public health challenges.

We acknowledge self-report survey data cannot be considered objective, our comparative analyses were not based on random or controlled allocation, and our conclusions are drawn from observed associations rather than causal attribution. Further, the interpretation of changes attributed to the interventions cannot rule out a Hawthorn effect, where awareness of being involved in research can independently lead to reported improvements in the outcomes being assessed [44]. The online, public health, opt-in design of ISLAND is subject to these methodological limitations, however the results remain important and defensible. Our application of the theory-informed mediation models strengthened the validity of results and has yielded valuable information about how and why differing motivational factors influence the success of the ISLAND public health approach to changing risk behaviours. The current study fits within a larger body of work planned for the ISLAND program. Next steps will investigate whether observed behaviour changes are sustained through to 2029, and if sustained low-risk health behaviours are associated with lower future biological and functional symptoms of dementia.

## Conclusion

The ISLAND framework and interventions, a personalised DRP report and the four-week PDMOOC, work synergistically to increase dementia risk knowledge and stimulate health behaviour change for dementia risk reduction. The DRP report appears to consistently support people to address their dementia risk factors. For those inclined to engage with it, engaging in formal education via the PDMOOC about how and why certain factors increase the risk of dementia is associated with stronger reductions in modifiable dementia risk factors than the DRP alone. Delivered within the ISLAND framework, both the DRP and the PDMOOC can be considered accessible and efficacious public health dementia prevention interventions. The online ISLAND framework can be implemented at scale, and offers a promising public health approach for supporting dementia prevention via health behaviour change at both the individual and aggregate-level.

### Electronic supplementary material

Below is the link to the electronic supplementary material.

Supplementary Material 1

## Data Availability

The deidentified datasets analysed for the current study (excluding cases where consent was provided only for use of data by the ISLAND research team) will be made available from the corresponding author on reasonable request.
