# Supplementary material for: An online, public health framework supporting behaviour change to reduce dementia risk: interim results from the ISLAND study linking ageing and neurodegenerative disease
Source: BMC Public Health. 2023 Sep 29;23:1886. doi: 10.1186/s12889-023-16805-2 (PMC10542675; doi:10.1186/s12889-023-16805-2)
Supplement: Supplementary file 1 — Supplementary Material 1 [file 12889_2023_16805_MOESM1_ESM.pdf]

Additional File 1: ISLAND Change – interim results

Contents

Table S1. Number of risk behaviours by time and number of exposures to the Dementia Risk Profile report....2

Table S2. Mean number of risk behaviours by time and number of exposures to the Dementia Risk Profile report, with and without engagement with the Preventing Dementia Massive Open Online Course....2

Table S3. Probability of moving into a different level of risk (low, medium, high) for each domain of the Dementia Risk Profile, from baseline to 2022....2

Table S4. Probability of moving into a different level of risk (low, medium, high) for each domain of the Dementia Risk Profile, by PDMOOC engagement....5

Supplementary Table S5: Descriptive summary data by timepoint for measures of dementia risk knowledge and motivations to change dementia risk behaviours....6

Sample r code for linear regression models....7

Sample r code for multinomial regression models....7

Sample r code for mediation analyses....8

Bartlett L, Bindoff A, Doherty K, Kim S, Eccleston C, Kitsos A, Roccati E, Alty J, King AE, Vickers JC. ***An online, public health framework supporting behaviour change to reduce dementia risk: interim results from the Island Study Linking Ageing and Neurodegenerative Disease.***

Submitted BMC Public Health March 2023. Accepted 21 September, 2023.
Find the published paper at https://doi.org/10.1186/s12889-023-16805-2

Table S1. Number of risk behaviours by time and number of exposures to the Dementia Risk Profile report.

Months	Exposures	EMMEAN	SE
0	1	2.164	0.022
12	2	1.745	0.020
24	3	1.650	0.019
36	4	1.601	0.024

EMMEAN and SE: estimated marginal means and standard errors from unadjusted linear mixed effects regression testing effects of the number of exposures to the personal Dementia Risk Profile report. Note: exposures also provide a proxy for time in the project, as the report is provided after completing baseline and then each annual survey.

Table S2. Number of risk behaviours by exposures to the Dementia Risk Profile report, with and without engagement with the Preventing Dementia Massive Open Online Course

Months	Exposures	PDMOOC	EMMEAN	SE
0	1	No	2.196	0.031
12	2	No	1.800	0.028
24	3	No	1.730	0.028
36	4	No	1.704	0.035
0	1	Yes	2.132	0.029
12	2	Yes	1.689	0.026
24	3	Yes	1.571	0.025
36	4	Yes	1.498	0.030

EMMEAN and SE: estimated marginal means and standard errors from unadjusted linear mixed effects regression testing effects of the number of exposures to the personal Dementia Risk Profile report (PDMOOC= no) and the effects following engagement following the PDMOOC. Note: exposures also provide a proxy for time in the project, as the report is provided after completing baseline and then each annual survey.

Table S3. Probability of moving into a different level of risk (low, medium, high) for each domain of the Dementia Risk Profile, from baseline to 2022

Risk factor	Baseline risk level	2022 risk level	Probability of change	SE	95%CI		t ratio	p value
Alcohol consumption								
	low	low	**0.828**	0.082	0.661	0.994	10.063	< 0.001
	low	medium	0.136	0.082	-0.030	0.302	1.656	0.106

	low	high	**0.009**	0.003	0.004	0.015	3.306	0.002
	medium	low	**0.270**	0.054	0.161	0.379	5.015	< 0.001
	medium	medium	**0.661**	0.054	0.551	0.771	12.188	< 0.001
	medium	high	**0.037**	0.007	0.023	0.050	5.385	< 0.001
	high	low	**0.106**	0.036	0.032	0.179	2.915	0.006
	high	medium	**0.529**	0.041	0.447	0.611	13.021	< 0.001
	high	high	**0.336**	0.025	0.285	0.388	13.228	< 0.001
Blood pressure management								
	low	low	**0.969**	0.005	0.960	0.978	208.072	< 0.001
	low	medium	**0.011**	0.002	0.006	0.016	4.568	< 0.001
	low	high	**0.019**	0.004	0.011	0.027	4.911	< 0.001
	medium	low	**0.828**	0.037	0.753	0.903	22.317	< 0.001
	medium	medium	**0.079**	0.027	0.025	0.133	2.940	0.005
	medium	high	**0.094**	0.031	0.032	0.155	3.064	0.004
	high	low	**0.835**	0.030	0.774	0.895	27.839	< 0.001
	high	medium	**0.054**	0.018	0.017	0.091	2.947	0.005
	high	high	**0.111**	0.027	0.057	0.165	4.159	< 0.001
Body mass index								
	low	low	**0.877**	0.041	0.793	0.961	21.156	< 0.001
	low	medium	**0.116**	0.041	0.032	0.199	2.794	0.008
	low	high	**0.007**	0.002	0.003	0.011	3.281	0.002
	medium	low	0.183	0.103	-0.025	0.391	1.779	0.083
	medium	medium	**0.757**	0.103	0.548	0.965	7.345	< 0.001
	medium	high	**0.061**	0.007	0.046	0.075	8.264	< 0.001
	high	low	0.040	0.049	-0.058	0.139	0.829	0.412
	high	medium	**0.442**	0.051	0.338	0.545	8.615	< 0.001
	high	high	**0.515**	0.016	0.481	0.548	31.213	< 0.001
Cholesterol management								
	low	low	**0.927**	0.028	0.870	0.984	32.949	< 0.001
	low	medium	**0.017**	0.003	0.012	0.023	6.064	< 0.001
	low	high	0.056	0.028	-0.001	0.112	1.990	0.054
	medium	low	**0.691**	0.091	0.507	0.875	7.600	< 0.001
	medium	medium	**0.129**	0.027	0.075	0.183	4.803	< 0.001
	medium	high	**0.180**	0.088	0.001	0.358	2.038	0.048
	high	low	**0.640**	0.103	0.433	0.848	6.236	< 0.001
	high	medium	**0.044**	0.009	0.026	0.062	4.863	< 0.001
	high	high	**0.316**	0.103	0.108	0.523	3.075	0.004
Cognitive activity								
	low	low	**0.927**	0.008	0.910	0.944	119.503	< 0.001
	low	high	**0.073**	0.008	0.056	0.090	9.393	< 0.001
	high	low	**0.701**	0.014	0.670	0.732	50.292	< 0.001
	high	high	**0.299**	0.014	0.268	0.330	21.436	< 0.001

Diabetes management							
low	low	**0.820**	0.086	0.647	0.994	9.576	< 0.001
low	medium	**0.012**	0.004	0.004	0.020	3.112	0.003
low	high	0.166	0.086	-0.007	0.339	1.939	0.060
medium	low	**0.658**	0.124	0.407	0.908	5.315	< 0.001
medium	medium	0.090	0.047	-0.005	0.186	1.910	0.063
medium	high	**0.252**	0.116	0.018	0.486	2.177	0.036
high	low	**0.536**	0.062	0.410	0.661	8.630	< 0.001
high	medium	0.013	0.007	0.000	0.026	1.984	0.054
high	high	**0.451**	0.062	0.326	0.577	7.289	< 0.001
Mediterranean diet adherence							
low	low	**0.692**	0.039	0.613	0.770	17.747	< 0.001
low	medium	**0.300**	0.039	0.221	0.379	7.710	< 0.001
low	high	0.000	0.000	0.000	0.000	0.991	0.328
medium	low	**0.234**	0.077	0.078	0.389	3.033	0.004
medium	medium	**0.613**	0.074	0.464	0.762	8.314	< 0.001
medium	high	0.153	0.089	-0.026	0.333	1.730	0.092
high	low	**0.000**	0.000	0.000	0.000	2.038	0.048
high	medium	**0.405**	0.040	0.324	0.487	10.053	< 0.001
high	high	**0.588**	0.040	0.507	0.670	14.614	< 0.001
Physical activity							
low	low	**0.979**	0.004	0.970	0.988	240.920	< 0.001
low	high	**0.021**	0.004	0.012	0.030	5.255	< 0.001
high	low	**0.821**	0.0267	0.762	0.890	30.810	< 0.001
high	high	**0.179**	0.027	0.120	0.238	6.712	< 0.001
Smoking							
low	low	**0.996**	0.001	0.994	0.999	834.259	< 0.001
low	medium	**0.002**	0.001	0.000	0.004	2.425	0.020
low	high	0.001	0.001	0.000	0.003	1.605	0.117
medium	low	**0.806**	0.050	0.705	0.907	16.096	< 0.001
medium	medium	**0.159**	0.043	0.072	0.246	3.685	< 0.001
medium	high	0.035	0.028	-0.021	0.092	1.269	0.212
high	low	**0.491**	0.058	0.373	0.609	8.445	< 0.001
high	medium	0.033	0.018	-0.003	0.069	1.837	0.074
high	high	**0.476**	0.060	0.354	0.598	7.895	< 0.001

Probability of Change: using multinomial logistic regression we estimated the probability of transition from risk state in baseline data (provided between October 2019 and June 2022) to risk state in October 2022; bold text denotes statistical significance with α=0.05. Results are averaged over PDMOOC, age, sex, education and socioeconomic status.

Please refer to the ISLAND Protocol paper for scoring criteria. http://dx.doi.org/10.2196/34688

Table S4. Probability of moving into a different level of risk (low, medium, high) for each domain of the Dementia Risk Profile, by PDMOOC engagement

Risk behaviour	PDMOOC exposed	2022 risk level	Probability of change	95%CI		p value	Effect of PDMOOC
**Alcohol consumption**							
	Yes - No	high	-0.003	-0.026	0.019	0.776	0.3% fewer
	Yes - No	medium	0.000	-0.036	0.036	0.997	0.0% more
	Yes - No	low	0.010	-0.023	0.043	0.547	1.0% more
**Blood pressure management**							
	Yes - No	high	-0.003	-0.023	0.017	0.772	0.3% fewer
	Yes - No	medium	-0.004	-0.021	0.012	0.581	0.4% fewer
	Yes - No	low	0.008	-0.015	0.031	0.508	0.8% more
**Body mass index**							
	Yes - No	high	-0.019	-0.040	0.003	0.094	1.9% fewer
	Yes - No	medium	0.002	-0.030	0.035	0.879	0.2% more
	Yes - No	low	0.016	-0.016	0.048	0.323	1.6% more
**Cholesterol management**							
	Yes - No	high	-0.041	-0.085	0.003	0.065	4.1% fewer
	Yes - No	medium	0.015	-0.006	0.036	0.150	1.5% more
	Yes - No	low	0.026	-0.020	0.073	0.258	2.6% more
**Cognitive activity**							
	Yes - No	low	-0.002	-0.027	0.024	0.890	0.2% fewer
	Yes - No	high	0.002	-0.024	0.027	0.890	0.2% more
**Diabetes management**							
	Yes - No	high	**-0.046**	-0.085	-0.006	0.024	**4.6% fewer**
	Yes - No	medium	0.007	-0.010	0.024	0.441	0.7% more
	Yes - No	low	0.039	-0.003	0.082	0.066	3.9% more
**Mediterranean diet adherence**							
	Yes - No	high	-0.038	-0.084	0.008	0.104	3.8% fewer
	Yes - No	medium	-0.004	-0.043	0.034	0.815	0.4% fewer
	Yes - No	low	**0.043**	0.011	0.075	0.010	**4.3% more**
**Physical activity**							
	Yes - No	low	0.025	-0.007	0.056	0.111	2.5% more
	Yes - No	high	-0.025	-0.056	0.007	0.111	2.5% fewer
**Smoking**							
	Yes - No	high	0.043	-0.003	0.088	0.064	4.3% more
	Yes - No	medium	**-0.047**	-0.081	-0.013	0.008	**4.7% fewer**
	Yes - No	low	0.005	-0.047	0.057	0.856	0.5% more

Results from multinomial models are averaged over age, sex, educational attainment and area-based Index of Relative Socio-economic Advantage and Disadvantage. Probability of change contrast analysis: PDMOOC engaged vs not; bold text denotes statistical significance with α=0.05. Please refer to the ISLAND Protocol paper for scoring criteria. http://dx.doi.org/10.2196/34688

Supplementary Table S5: Descriptive summary data by timepoint for measures of dementia risk knowledge and motivations to change dementia risk behaviours

Outcome variable	BL	Oct-20	Oct-21	Oct-22
**Dementia risk literacy**	n=3038	n=2010	n=2136	n=3001
General knowledge				
Mean (SD)	12.4 (4.05)	13.9 (4.13)	13.6 (4.20)	13.8 (4.15)
Median [Min, Max]	12.0 [1.00, 22.0]	13.0 [2.00, 22.0]	13.0 [0, 22.0]	13.0 [2.00, 22.0]
Correct attributions				
Mean (SD)	12.0 (5.14)	13.9 (4.63)	18.0 (5.96)	18.2 (6.18)
Median [Min, Max]	11.0 [0, 28.0]	13.0 [0, 20.0]	17.0 [0, 28.0]	18.0 [0, 28.0]
Misconceptions				
Mean (SD)	1.94 (2.37)	2.89 (2.81)	2.90 (2.82)	2.59 (2.74)
Median [Min, Max]	1.00 [0, 12.0]	2.00 [0, 12.0]	2.00 [0, 12.0]	2.00 [0, 12.0]
**Motivations to change dementia risk behaviours**	n=3038	n=1972	n=2125	n=2987
Susceptibility				
Mean (SD)	10.8 (2.74)	10.3 (2.74)	10.5 (2.77)	10.5 (2.81)
Median [Min, Max]	11.0 [4.00, 20.0]	10.0 [4.00, 20.0]	11.0 [4.00, 20.0]	11.0 [4.00, 20.0]
Severity				
Mean (SD)	15.5 (3.01)	15.1 (3.05)	15.2 (2.95)	15.3 (3.03)
Median [Min, Max]	15.0 [5.00, 25.0]	15.0 [5.00, 25.0]	15.0 [5.00, 25.0]	15.0 [5.00, 25.0]
Benefits				
Mean (SD)	15.0 (1.92)	15.2 (2.03)	15.2 (2.05)	15.2 (2.05)
Median [Min, Max]	15.0 [8.00, 20.0]	15.0 [5.00, 20.0]	15.0 [6.00, 20.0]	15.0 [4.00, 20.0]
Barriers				
Mean (SD)	8.21 (2.26)	8.31 (2.26)	8.43 (2.25)	8.59 (2.41)
Median [Min, Max]	8.00 [4.00, 20.0]	8.00 [4.00, 18.0]	8.00 [4.00, 17.0]	8.00 [4.00, 20.0]
Cues to action				
Mean (SD)	13.0 (2.38)	13.0 (2.44)	12.9 (2.39)	13.1 (2.43)
Median [Min, Max]	13.0 [4.00, 20.0]	13.0 [4.00, 20.0]	13.0 [4.00, 20.0]	13.0 [4.00, 20.0]
Health motivation				
Mean (SD)	15.4 (2.09)	15.3 (2.08)	15.2 (2.11)	15.3 (2.14)
Median [Min, Max]	15.0 [7.00, 20.0]	15.0 [8.00, 20.0]	15.0 [8.00, 20.0]	15.0 [6.00, 20.0]
Self-efficacy				
Mean (SD)	7.72 (1.17)	7.79 (1.19)	7.75 (1.19)	7.70 (1.22)
Median [Min, Max]	8.0 [3.0, 10.0]	8.0 [2.0, 10.0]	8.0 [2.0, 10.0]	8.0 [2.0, 10.0]

BL: baseline completed between Oct 2019 and June 2022; Oct-20: data from sample with BL prior to August 2020 and 2020 survey data; Oct-21: data from sample with BL prior to August 2021 and 2021 survey data; Oct-22: data from sample with BL prior to August 2022 and survey data in 2022. Dementia Risk Literacy measured using the Knowledge of Dementia Risk Reduction scale[1]; Motivations to change dementia risk behaviours measured using the Motivations the Change Lifestyle and Behaviours for Dementia Risk Reduction scale [2]

Sample r code for linear regression models

Estimate direction and magnitude of associations between intervention exposures and outcome variables (sample code shows DRP change)

```
#unadjusted model (no mooc)

m0 <- lmer(DRP_total_all ~  exposures + log(t_months+1) + (1+exposures|UID), data = d)

#unadjusted model - effect of mooc

m1 <- lmer(DRP_total_all ~ exposures*mooc + log(t_months+1) + (1+exposures|UID), data = d)

# covariate adjusted model

m2 <- lmer(DRP_total_all ~ exposures*mooc +
                log(t_months+1) +
                age +
                gender +
                BACKGROUND_marital_cat  +
                BACKGROUND_education_cat +
                IRSAD_Decile
            + (1+exposures|UID), data = d)
```

Sample r code for multinomial regression models

Estimate matrix of transition probabilities across risk levels in each DRP domain, with and without contrast for intervention exposures. (Sample shows code for alcohol tests.)

Packages: lmerTest3 lme44 and emmeans5

#Calculate the omnibus statistic for the change from BL to Oct22 (participation in ISLAND, including first exposure to DRP)

[1] Eccleston C, Kitsos A, Doherty K. Assessing dementia risk reduction knowledge: development of the KoDeRR instrument (Conference Paper). In: Alzheimer's Disease International, editor. 35th Global Conference of Alzheimer's Disease International: New horizons in dementia. London 2022.

[2] Kim S, Sargent-Cox K, Cherbuin N, Anstey KJ. Development of the motivation to change lifestyle and health behaviours for dementia risk reduction scale. Dementia and Geriatric Cogntive Disorders Extra. 2014;4(2):172-83.

[3] Kuznetsova A, Brockhoff PB, Christensen RHB. lmerTest Package: Tests in Linear Mixed Effects Models. Journal of Statistical Software. 2017;82(13):1 - 26. 10.18637/jss.v082.i13

[4] Bates D, Mächler M, Bolker B, Walker S. Fitting Linear Mixed-Effects Models Using lme4. Journal of Statistical Software. 2015;67(1):1 - 48. 10.18637/jss.v067.i01

[5] Searle SR, Speed FM, Milliken GA. Population Marginal Means in the Linear Model: An Alternative to Least Squares Means. The American Statistician. 1980;34(4):216-21. 10.1080/00031305.1980.10483031

```
a <- anova(m2, multinom(alc_Oct22 ~ mooc + age + gender + BACKGROUND_education_cat + IRSAD_Decile, data = w %>%
        filter(!is.na(alc_BL)), Hess = TRUE, trace = FALSE))

a1 <- a[2,] %>% mutate(stat = round(`LR stat.`), prob = case_when(`Pr(Chi)` == 0 ~ "< 0.001",
        `Pr(Chi)` < 0.001 ~ "< 0.001",
        TRUE ~ format(round(`Pr(Chi)`, 3), nsmall = 3)))
```

```
## adjusted model

m2 <- multinom(alc_Oct22 ~ alc_BL + mooc + age + gender + BACKGROUND_education_cat + IRSAD_Decile, data = w, Hess = TRUE, trace = FALSE)

### contrast – bl to 2022 - mooc not included

em <- emmeans(m2, pairwise ~ alc_Oct22|alc_BL, at = list(alc_BL = c('low', 'medium', 'high')))

em1 <- as.data.frame(em$emmeans)

em_p <- as.data.frame(test(em$emmeans))

em2 <- left_join(em1, em_p)

probabilities_NO_MOOC <- em2 %>%
        magrittr::set_names(c("October 2022 Risk", "Baseline Risk", "prob", "SE", "df", "lower_CI", "upper_CI", "t_ratio", "p_value")) %>%
            mutate(phenotype = "alcohol")
```

Sample r code for mediation analyses

####Estimate the mediating role of process variables (knowledge, motivations) on change in DRP by PDMOOC engagement. (Sample code uses the misconceptions dimension from the Knowledge of Dementia Risk Reduction (KoDERR) scale.)

Packages = lavaan[6]

```
# Model 1: Tested the mediating role of knowledge on the path between PDMOOC and DRP.

m1 <- 'DRP_z_Oct22 ~ DRP_z_BL + c1*mooc + b1*knowledge_Oct22

   KODERR_Oct22 ~ knowledge_BL + a1*mooc

   ind := a1*b1

   pmed := a1*b1/(c1 + a1*b1)'

# fit a "full information maximum likelihood" SEM

fit1 <- sem(m1, data = w, meanstructure = TRUE, se = 'bootstrap', missing = 'ml', bootstrap = 5000L)
```

[6] Rosseel Y. lavaan: An R Package for Structural Equation Modeling. Journal of Statistical Software. 2012;48(2):1-36.
